# Case report: Identification of a recurrent pathogenic *DHDDS* mutation in Chinese family with epilepsy, intellectual disability and myoclonus

**DOI:** 10.3389/fgene.2023.1208540

**Published:** 2023-10-10

**Authors:** Yi Dong, Yi Zhang, Yue Sheng, Fang Wang, Lv Liu, Liang-Liang Fan

**Affiliations:** ^1^ Department of Cell Biology, School of Life Sciences, Central South University, Changsha, China; ^2^ Medical Psychological Center, Medical Psychological Institute of Central South University, National Clinical Research Center for Mental Disorders, The Second Xiangya Hospital of Central South University, Changsha, China; ^3^ Department of Endocrinology, The Third Xiangya Hospital of Central South University, Changsha, Hunan, China; ^4^ Department of Respiratory Medicine, Clinical Center for Gene Diagnosis and Therapy, Diagnosis and Treatment Center of Respiratory Disease, Diagnosis and Treatment Center of Respiratory Disease, The Second Xiangya Hospital of Central South University, Changsha, China

**Keywords:** epilepsy, myoclonus, *DHDDS*, mutation, whole exome sequencing

## Abstract

**Background:** Heterozygous mutations in the *dehydrodolichol diphosphate synthase* (*DHDDS*) gene are one of the causes generating developmental and epileptic encephalopathies. So far, only eleven mutations in the DHDDS gene have been identified. The mutation spectrum of the *DHDDS* gene in the Chinese population remains unclear.

**Methods:** In this study, we enrolled a Chinese family with myoclonus and/or epilepsy and intellectual disability. The epilepsy and myoclonic tremor were improved after deep brain stimulation (DBS) of the subthalamic nucleus (STN) treatment. Whole exome sequencing and Sanger sequencing were employed to explore the genetic variations of the family.

**Results:** Subsequent to data filtering, we identified a recurrent pathogenic mutation (NM_001243564.1, c.113G>A/p.R38H) in the *DHDDS* gene in the proband. Sanger sequencing further validated that the presence of the mutation in his affected mother but absent in the health family members. Further bioinformatics analysis revealed that this mutation (p.R38H), located in an evolutionarily conserved region of DHDDS, was predicted to be deleterious.

**Discussion:** In this report, we present the first case of intractable epilepsy and/or myoclonus caused by p.R38H mutation of the *DHDDS* gene in the Chinese population. Furthermore, this study represents the third report of autosomal dominant familial inheritance of *DHDDS* mutation worldwide.

## Introduction

Dehydrodolichol diphosphate synthase (DHDDS) is an enzyme crucial for the synthesis of dolichol monophosphate and for global N-linked glycosylation (Cunillera et al., 2000; Galosi et al., 2022). The gene encoding the DHDDS protein is located at 1p36.11 and was first identified as a disease-causing gene of retinitis pigmentosa, a recessive genetic disorder (Zelinger et al., 2011). However, in 2017, Hamdan et al. (2017) reported that heterozygous mutations in the *DHDDS* gene were responsible for developmental and epileptic encephalopathies (DEE). In addition, several other genes involved in the dolichol-dependent protein glycosylation pathway have also been identified, including *NUS1 Dehydrodolichyl Diphosphate Synthase Subunit* (*NUS1*) and *ALG10 Alpha-1,2-Glucosyltransferase* (*ALG10*), which are also disease-causing genes for epilepsy (Araki et al., 2020; Courage et al., 2021). At present, approximately 11 *DHDDS* mutations have been reported in 39 patients, resulting in various types of movement disorders, epilepsy, or other symptoms (Kim et al., 2021b; Galosi et al., 2022; Jiao et al., 2022). Most of these variants have been confirmed to be *de novo* events, except for two mutations (p.R205Q and p.R211Q) that have been reported in two families (Wood et al., 2021; Galosi et al., 2022). However, no family history-related *DHDDS* mutations have been reported in the Chinese population, and the relationship between different phenotypes and *DHDDS* mutations remains unclear.

## Case presentation

**FIGURE 1:**
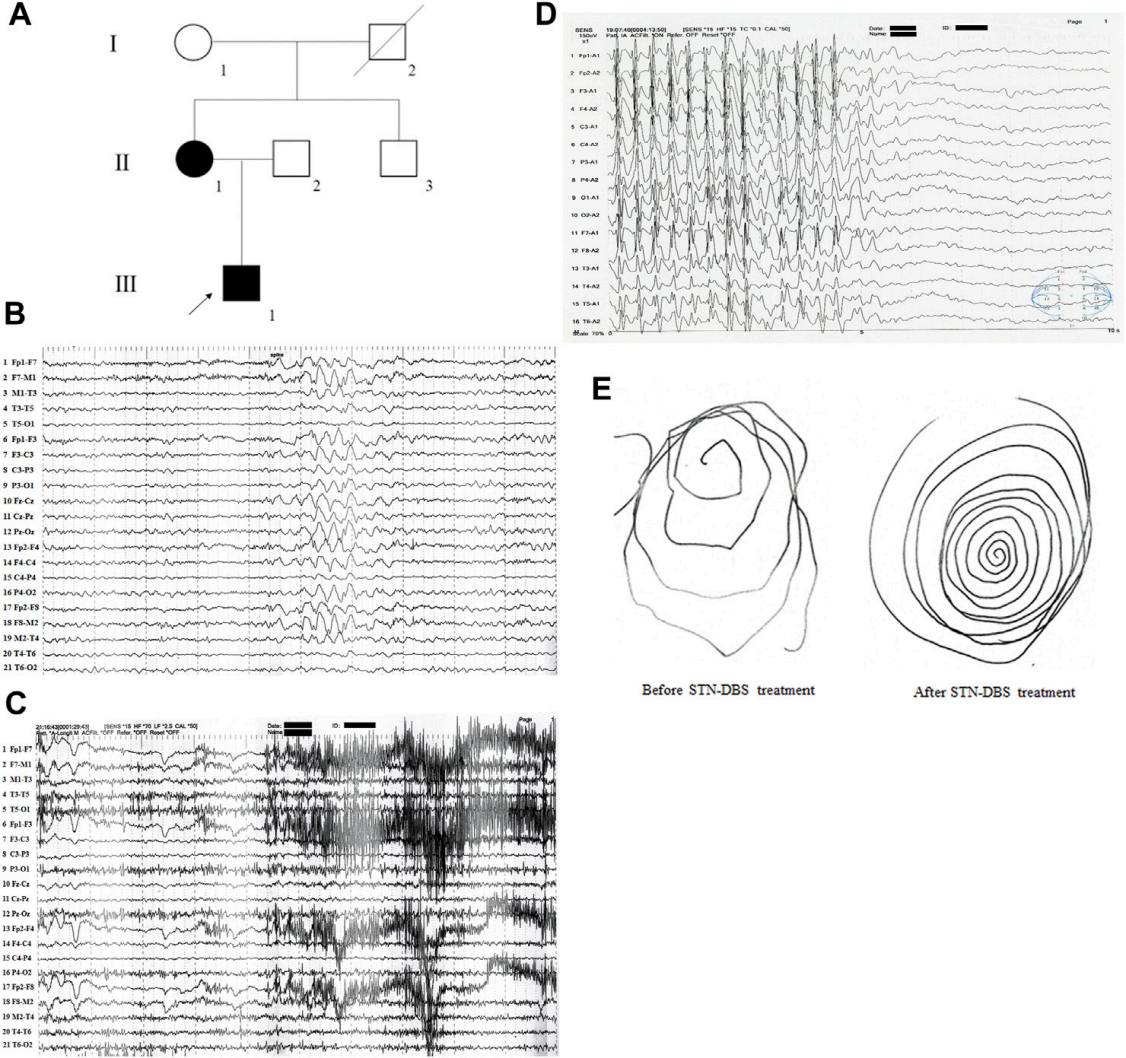
The clinical data of the family with epilepsy and/or myoclonus. **(A)** Pedigree figure. Family members are identified by generations and numbers. Squares indicate male family members; circles, female members; closed symbols, the affected members; open symbols, unaffected members; arrow, proband. **(B)** 24 h of video scalp EEG monitoring showed diffuse spike waves during the interictal period. **(C)** Synchronized surface EMG showed spikes when the patient has myoclonic seizures. **(D)** Synchronized surface EEG showed spikes when the patient has myoclonic seizures. **(E)** Patient’s handwriting showed tremor-like movement before and after STN-DBS treatment.

A two-generation Han Chinese pedigree, comprising six members, was recruited for this study (Figure 1A). The proband (III-1), a 28-year-old male, exhibited mild intellectual disability and was born to non-consanguineous Chinese parents following an uneventful full-term pregnancy. At the age of 3 years, he began to display mild intentional tremors in both hands. By the age of eight, he began experiencing absence seizures, and the myoclonus in his hands worsened with age, gradually extending to the lower extremities, head, and trunk. On neurological examination, generalized myoclonic tremors were detected at rest and were provoked by action (Supplementary Video S1, after deep brain stimulation (DBS) of the subthalamic nucleus (STN) treatment). Scalp electroencephalogram (EEG) monitoring conducted over a 24-h period revealed diffuse spike waves during the interictal period (Figure 1B). The myoclonic status was monitored during the ictal and awake phases, accumulating in the eyelids, neck muscles, bilateral upper extremities, and trunk. Spikes (deltoid and thenar muscles) were observed on synchronized surface electromyography (EMG) recordings (Figure 1C), and epileptiform discharges linked to EMG spikes were found on synchronized EEG recordings (Figure 1D). The patient was treated with sodium valproate, followed by lamotrigine, levetiracetam, clonazepam, and lorazepam; however, all had poor effects. Consequently, the patient underwent STN-DBS in 2020. The myoclonic tremor symptoms in this patient improved postoperatively, and the epilepsy was well controlled with the above-mentioned antiepileptic drugs (Figure 1E). Further investigation of family history revealed that the mother of the proband had a history of head tremors, whereas no other neurological disorders were observed in the other family members.

**FIGURE 2:**
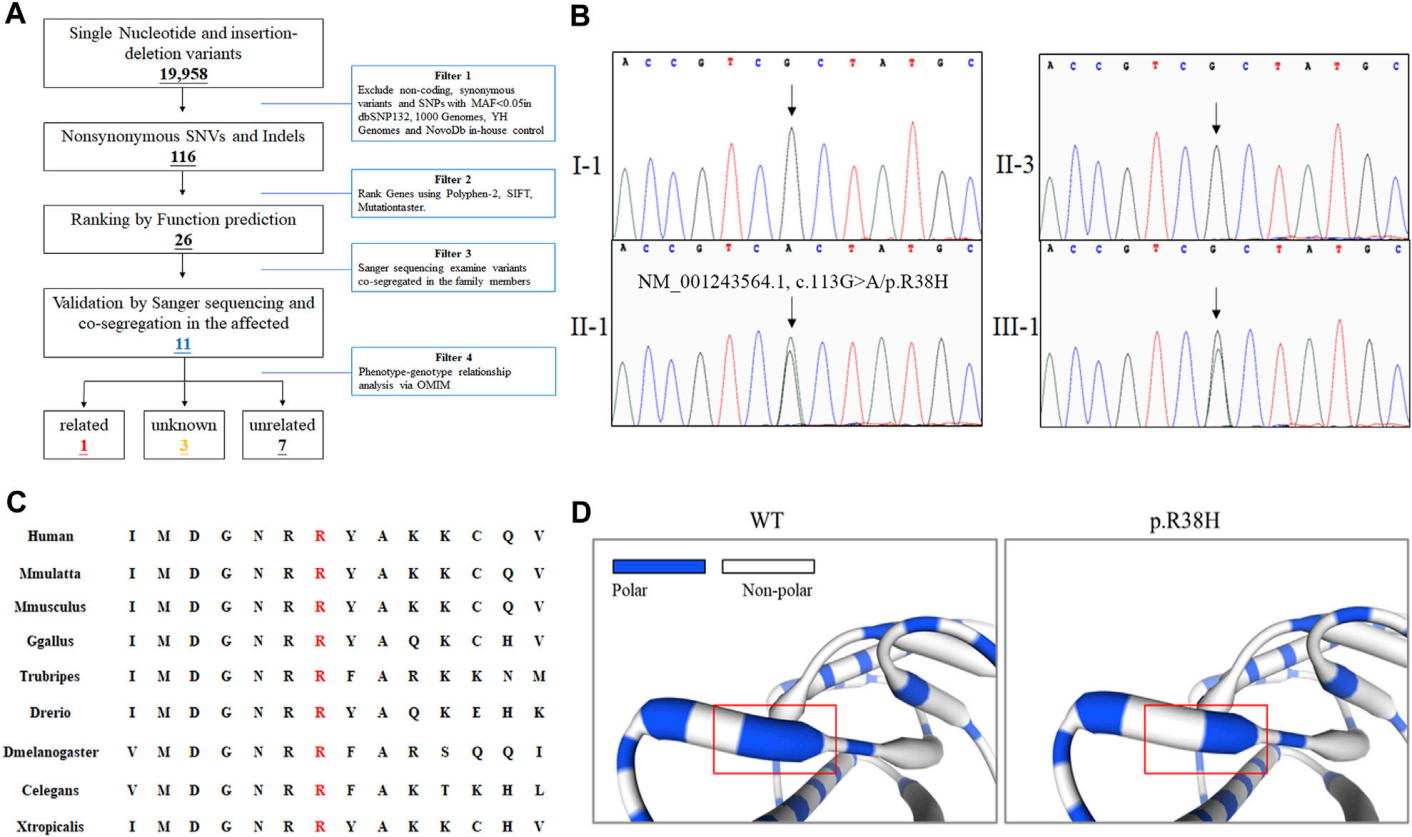
The genetic analysis of family. **(A)** Schematic representation of the filter strategies employed in our study. **(B)** Sanger sequencing of DHDDS confirmed the mutation (NM_001243564.1, c.113G>A/p. R38H) in II-1 and III-1. Arrow indicates the c.113G site. **(C)** Alignment of multiple DHDDS protein sequences across species. The R38H affected amino acid locates in the highly conserved amino acid region in different mammals (from Ensembl). Letters in red show the R38 site. **(D)** The part structure of WT and mutated DHDDS protein. Red squares represent R38 site.

The proband was subjected to whole-exome sequencing, as previously described (Fan et al., 2018). The mean coverage of the target regions obtained for the proband was 99.8%, with an average sequencing depth of × 90.21. A total of 9,683 single nucleotide polymorphisms and 10,275 indels were identified. After data filtering (Figure 2A), 11 mutations were detected following public database filtering, bioinformatic program prediction, and family cosegregation analysis (Table 1). We then analyzed the evolutionary conservation (GERP) and the relationship between the candidate gene and phenotypes (OMIM) and found that the rare mutation (NM_001243564.1, c.113G>A/p. R38H) of *DHDDS* has a high possibility to be the genetic lesion of the family. Sanger sequencing confirmed that this mutation was present in the proband and his mother but absent in his father (Figure 2B). This rare mutation resulted in the substitution of arginine with histidine and was located at a highly evolutionarily conserved site (Figure 2C). This was not found in our local control cohort of 200 individuals and Genome Aggregation Database (gnomAD) database. Furthermore, a protein structure model constructed using the Swiss model revealed that the mutation (NM_001243564.1, c.113G>A/p. R38H) altered the polarity of the protein, which may further affect the protein function of DHDDS (Figure 2D). According to American College of Medical Genetics and Genomics guidelines (Richards et al., 2015), the mutation met the pathogenic criteria (PS1+PM1+PM2+PP1+PP2).

**TABLE 1:** The gene list of Sanger sequencing validation and co-segregation analysis.

Gene	Variant	Genotype	SIFT	Polyphen-2	Mutation taster	MAF in genomAD	GERP	Diseases in OMIM
DHDDS	NM_001243564.1:c.113G>A:p.R38H	het	D	D	D	-	5.72	AR, retinitis pigmentosa 59; AD, developmental delay and seizures
APH1A	NM_001077628.2:c.2T>C	het	D	D	D	-	4.86	alzheimer disease
ZNF638	NM_001014972.2: c.351A>T:p.K117N	het	D	D	D	-	0.554	cutaneous t cell lymphoma
TMEM30A	NM_001143958.1:c.867G>T:p.L289F	het	D	D	D	-	4	intrahepatic cholestasis
ABCC2	NM_000392.4:c.4384G>A:p.E1462K	het	D	D	D	0.02	4.98	AR, intrahepatic cholestasis
EFS	NM_005864.3:c.196_19delinsCACCAGG	het	-	-	-	-	-	ocular hyperemia
CARHSP1	NM_001042476.2:c.169G>T:p.A57S	het	D	D	D	-	5.46	-
ALOXE3	NM_001165960.1: c.1711C>T:p.P571S	het	D	D	D	-	4.46	AR, ichthyosis
EPG5	NM_020964.2: c.7725G>T:p.L2575F	het	D	D	D	-	6.07	AR, spastic paraplegia 49
MED16	NM_005481.2: c.602C>G:p.T201R	het	D	D	D	-	2.31	-
ARID3A	NM_005224.2: c.380G>T:p.W127L	het	D	D	D	-	3.75	-

D, deleterious; AD, autosomal dominant; AR, autosomal recessive; MAF, minor allele frequency.

## Discussion

Currently, only 11 *DHDDS* mutations, identified in 39 patients, have been reported to result in DEE (Kim et al., 2021b; Galosi et al., 2022). Most patients were from Europe and North America, whereas 14 were from Eastern Asia (Togashi et al., 2020; Kim et al., 2021a; Jiao et al., 2022). In this study, we identified a rare mutation (NM_001243564.1, c.113G>A/p. R38H) of *DHDDS* in a Han Chinese family. This is the second report of DHDDS-related epilepsy in China and the third report of autosomal dominant familial inheritance of *DHDDS* mutation worldwide. Our findings further support that DHDDS play a crucial role in the occurrence and development of epilepsy.

Among the 39 patients with *DHDDS* mutations (Table 2), most presented with early onset epilepsy (0.5–8 years), and only nine showed late-onset epilepsy. Most of these patients exhibited different degrees of intellectual disability, generalized tonic-clonic seizures, and movement disorders, including tremor, dystonia, ataxia, and myoclonus. Interestingly, different phenotypes were associated with the DHDDS domain in which the mutations were located. The missense variant (NM_001243564.1, c.113G>A/p. R38H) in *DHDDS* was first reported in a patient with developmental and epileptic encephalopathy in an Epi4K study (Epi et al., 2013). Hamdan et al. subsequently identified *de novo* missense *DHDDS* mutations (NM_001243564.1, c.110G>A/p. R37H) that lie adjacent to p. R38H, and the defined *DHDDS* was found to be responsible for the DEE [4]. In our case, the proband also showed early onset epilepsy, intellectual disability, and myoclonus, consistent with most of the other reported cases (Kim et al., 2021b; Galosi et al., 2022).

**TABLE 2:** Clinical features of DHDDS-related epilepsy.

	c.104G>A p.G35E	c.109C>T p.R37C	c.110G>A p.R37H	c.113G>A p.R38H	c.113G>C p.R38P	c.124_126del p.K42del	c.283G>A p.D95N	c.614G>A p.R205Q	c.632G>A p.R211Q	c.638G>A p.S213N	c.698C>G p.P233R	All variants
Number of cases (%)	2	6	8	**1**	1	1	1	4	13	1	1	39
Intellectual disability	2 (100%)	6 (100%)	8 (100%)	**1(100%)**	1 (100%)	1 (100%)	-	3 (75%)	11 (85%)	1 (100%)	1 (100%)	35 (90%)
Epilepsy												
Epilepsy onset at 0.5–8 years	2 (100%)	6 (100%)	5 (63%)	**1(100%)**	1 (100%)	1 (100%)	-	2 (50%)	9 (69%)	1 (100%)	-	28 (72%)
Generalized tonic clonic	1 (50%)	5 (83%)	3 (38%)	**1(100%)**	1 (100%)	1 (100%)	-	2 (50%)	6 (46%)	1 (100%)	-	21 (54%)
Myoclonic	1 (50%)	3 (50%)	6 (75%)	**1(100%)**	-	1 (100%)	-	2 (50%)	5 (38%)	-	-	19 (49%)
Febrile myoclonic	-	2 (33%)	4 (50%)	**-**	-	-	1 (100%)	3 (75%)	1 (7%)	-	-	11 (28%)
Absence with eyelid myoclonia	-	-	1 (13%)	**1(100%)**	-	-	-	2 (50%)	2 (15%)	-	-	6 (15%)
Atypical absences	-	1 (25%)	3 (38%)	**-**	-	1 (100%)	-	-	2 (15%)	1 (100%)	-	8 (21%)
Movement disorder												
Tremor	2 (100%)	4 (67%)	3 (38%)	**1(100%)**	1 (100%)	1 (100%)	1 (100%)	3 (75%)	9 (69%)	1 (100%)	-	26 (67%)
Myoclonus	1 (50%)	4 (67%)	1 (13%)	**1(100%)**	-	1 (100%)	1 (100%)	3 (75%)	6 (46%)	1 (100%)	1 (100%)	20 (51%)
Ataxia	2 (100%)	5 (83%)	4 (50%)	**-**	1 (100%)	-	1 (100%)	3 (75%)	8 (62%)	1 (100%)	-	25 (64%)
Dystonia	1 (50%)	1 (17%)	-	**-**	-	-	-	2 (50%)	3 (23%)	-	-	7 (18%)
Parkinsonism	-	-	1 (13%)	**-**	-	1 (100%)	-	-	5 (38%)	1 (100%)	-	8 (21%)
Chorea	1 (50%)	1 (17%)	-	**-**	-	-	-	-	-	-	1 (100%)	3 (8%)
Stereotypic movements	-	2 (33%)	1 (13%)	**-**	-	-	-	-	-	-	-	3 (8%)

The bold values means the mutation identified in this study.

*DHDDS* encodes a protein involved in dolichol biosynthesis on the cytoplasmic face of the endoplasmic reticulum (ER) (van Duijn et al., 1986). Previous studies have suggested that dolichol plays a crucial role in membrane trafficking, particularly between the ER and lysosomal-endosomal system, which is critical for brain development (Wu et al., 2017; Stojkovska et al., 2022). In addition, DHDDS can form a complex with the NUS1-encoded Nogo-B receptor (NgBR) (Bar-El et al., 2020), another key gene involved in neurodegeneration (Xue et al., 2022), which can further regulate protein glycosylation (Edani et al., 2020). In this study, the recurrent pathogenic mutation (NM_001243564.1, c.113G>A/p. R38H) of *DHDDS* altered the polarity of the protein, which may further disrupt the function of DHDDS protein.

In our study, the proband accepted STN-DBS treatment, which is usually intended for the appropriate treatment of a movement disorders. In recent years, STN-DBS has become a promising option for patients with epilepsy and appropriate indications for movement disorders, particularly for drug-refractory epilepsy (Valentin et al., 2013). Analogously, in our case, the patient’s epilepsy symptoms cannot be controlled after treatment with five different antiepileptic drugs. Hence, the patient accepted the STN-DBS treatment. After STN-DBS, the epilepsy of the patient was well controlled, and his myoclonic tremor symptoms improved. Our study further confirmed that STN-DBS surgery is an effective therapeutic regimen for epilepsy and an appropriate indication for movement disorders. Furthermore, our study also provided a viable treatment option for patients harboring *DHDDS* mutations.

Most patients (36/39) with *DHDDS* mutations have been validated for *de novo* events (Kim et al., 2021b; Galosi et al., 2022). Only two mutations (p.R205Q and p.R211Q) were found to have an autosomal-dominant familial inheritance pattern (Wood et al., 2021; Galosi et al., 2022). The mother of the proband carries a rare mutation (NM_001243564.1; c.113G>A/p. R38H) of *DHDDS*, presented only with tremors, and no other neurological disorders were detected, consistent with a previously reported family carrying the p.R205Q mutation, whereas the father of the proband, who carried the p.R211Q mutation, showed no similar symptoms. At present, the variable expression of epilepsy genes is known, and some cases could be carriers and show only EEG abnormalities. Mosaic carriers may explain this phenomenon (Robens et al., 2022). Here, we reported another DHDDS mutation which presented with autosomal dominant inheritance pattern and lead to intractable epilepsy and/or myoclonus.

## Conclusion

In conclusion, by employing whole exome sequencing and Sanger sequencing, we identified a rare mutation (NM_001243564.1, c.113G>A/p. R38H) of *DHDDS* in a Chinese family. We may report the first case of intractable epilepsy and/or myoclonus caused by p.R38H mutation of *DHDDS* gene in the Chinese population. This study was also the third report of autosomal dominant familial inheritance of *DHDDS* mutation worldwide.

## Data Availability

The raw data supporting the conclusion of this article will be made available by the authors, without undue reservation.
