# Supplementary material for: Case report: Identification of a recurrent pathogenic DHDDS mutation in Chinese family with epilepsy, intellectual disability and myoclonus
Source: Front Genet. 2023 Oct 10;14:1208540. doi: 10.3389/fgene.2023.1208540 (PMC10597645; doi:10.3389/fgene.2023.1208540)
Supplement: Supplementary file 1 [file Table1.docx]

Table 1. The gene list of Sanger sequencing validation and co-segregation analysis

| Gene | Variant | Genotype | | SIFT | Polyphen-2 | Mutation  Taster | MAF in genomAD | GERP | Diseases in OMIM |
| --- | --- | --- | --- | --- | --- | --- | --- | --- | --- |
| DHDDS | NM_001243564.1:c.113G>A:p.R38H | | het | D | D | D | - | 5.72 | AR, retinitis pigmentosa 59; AD, developmental delay and seizures |
| APH1A | NM_001077628.2:c.2T>C | | het | D | D | D | - | 4.86 | alzheimer disease |
| ZNF638 | NM_001014972.2: c.351A>T:p.K117N | | het | D | D | D | - | 0.554 | cutaneous t cell lymphoma |
| TMEM30A | NM_001143958.1:c.867G>T:p.L289F | | het | D | D | D | - | 4 | intrahepatic cholestasis |
| ABCC2 | NM_000392.4:c.4384G>A:p.E1462K | | het | D | D | D | 0.02 | 4.98 | AR, intrahepatic cholestasis |
| EFS | NM_005864.3:c.196_19delinsCACCAGG | | het | - | - | - | - | - | ocular hyperemia |
| CARHSP1 | NM_001042476.2:c.169G>T:p.A57S | | het | D | D | D | - | 5.46 | - |
| ALOXE3 | NM_001165960.1: c.1711C>T:p.P571S | | het | D | D | D | - | 4.46 | AR, ichthyosis |
| EPG5 | NM_020964.2: c.7725G>T:p.L2575F | | het | D | D | D | - | 6.07 | AR, spastic paraplegia 49 |
| MED16 | NM_005481.2: c.602C>G:p.T201R | | het | D | D | D | - | 2.31 | - |
| ARID3A | NM_005224.2: c.380G>T:p.W127L | | het | D | D | D | - | 3.75 | - |

D, deleterious; AD, autosomal dominant; AR autosomal recessive; MAF, Minor Allele Frequency.
