# Supplementary material for: Case report: Identification of a recurrent pathogenic DHDDS mutation in Chinese family with epilepsy, intellectual disability and myoclonus
Source: Front Genet. 2023 Oct 10;14:1208540. doi: 10.3389/fgene.2023.1208540 (PMC10597645; doi:10.3389/fgene.2023.1208540)
Supplement: Supplementary file 2 [file Table2.DOCX]

Table 2. Clinical features of DHDDS-related epilepsy.

|  | c.104G>A p.G35E | c.109C>T p.R37C | c.110G>A p.R37H | **c.113G>A p.R38H** | c.113G>C p.R38P | c.124_126del p.K42del | c.283G>A p.D95N | c.614G>A p.R205Q | c.632G>A p.R211Q | c.638G>A p.S213N | c.698C>G p.P233R | All variants |
| --- | --- | --- | --- | --- | --- | --- | --- | --- | --- | --- | --- | --- |
| Number of cases (%) | 2 | 6 | 8 | **1** | 1 | 1 | 1 | 4 | 13 | 1 | 1 | 39 |
| Intellectual disability | 2(100%) | 6(100%) | 8(100%) | **1(100%)** | 1(100%) | 1(100%) | - | 3(75%) | 11(85%) | 1(100%) | 1(100%) | 35(90%) |
| Epilepsy |  |  |  |  |  |  |  |  |  |  |  |  |
| Epilepsy onset at 0.5-8 years | 2(100%) | 6(100%) | 5(63%) | **1(100%)** | 1(100%) | 1(100%) | - | 2(50%) | 9(69%) | 1(100%) | - | 28(72%) |
| Generalized tonic clonic | 1(50%) | 5(83%) | 3(38%) | **1(100%)** | 1(100%) | 1(100%) | - | 2(50%) | 6(46%) | 1(100%) | - | 21(54%) |
| Myoclonic | 1(50%) | 3(50%) | 6(75%) | **1(100%)** | - | 1(100%) | - | 2(50%) | 5(38%) | - | - | 19(49%) |
| Febrile myoclonic | - | 2(33%) | 4(50%) | **-** | - | - | 1(100%) | 3(75%) | 1(7%) | - | - | 11(28%) |
| Absence with eyelid myoclonia | - | - | 1(13%) | **1(100%)** | - | - | - | 2(50%) | 2(15%) | - | - | 6(15%) |
| Atypical absences | - | 1(25%) | 3(38%) | **-** | - | 1(100%) | - | - | 2(15%) | 1(100%) | - | 8(21%) |
| Movement disorder: |  |  |  |  |  |  |  |  |  |  |  |  |
| Tremor | 2(100%) | 4(67%) | 3(38%) | **1(100%)** | 1(100%) | 1(100%) | 1(100%) | 3(75%) | 9(69%) | 1(100%) | - | 26(67%) |
| Myoclonus | 1(50%) | 4(67%) | 1(13%) | **1(100%)** | - | 1(100%) | 1(100%) | 3(75%) | 6(46%) | 1(100%) | 1(100%) | 20(51%) |
| Ataxia | 2(100%) | 5(83%) | 4(50%) | **-** | 1(100%) | - | 1(100%) | 3(75%) | 8(62%) | 1(100%) | - | 25(64%) |
| Dystonia | 1(50%) | 1(17%) | - | **-** | - | - | - | 2(50%) | 3(23%) | - | - | 7(18%) |
| Parkinsonism | - | - | 1(13%) | **-** | - | 1(100%) | - | - | 5(38%) | 1(100%) | - | 8(21%) |
| Chorea | 1(50%) | 1(17%) | - | **-** | - | - | - | - | - | - | 1(100%) | 3(8%) |
| Stereotypic movements | - | 2(33%) | 1(13%) | **-** | - | - | - | - | - | - | - | 3(8%) |
